# Monocyte/High-Density Lipoprotein Ratio Predicts the Prognosis of Large Artery Atherosclerosis Ischemic Stroke

**DOI:** 10.3389/fneur.2021.769217

**Published:** 2021-11-29

**Authors:** Youyu Li, Daqing Chen, Laifang Sun, Zhibo Chen, Weiwei Quan

**Affiliations:** ^1^Department of Emergency Medicine, The Second Affiliated Hospital and Yuying Children's Hospital of Wenzhou Medical University, Wenzhou, China; ^2^Department of Neurology, The First Affiliated Hospital of Wenzhou Medical University, Wenzhou, China

**Keywords:** monocyte, high density lipoprotein, prognosis, large artery atherosclerosis, ischemic stroke

## Abstract

**Objective:** Monocyte to high-density lipoprotein ratio is considered as a new inflammatory marker and has been used to predict the severity of coronary heart disease and the incidence of adverse cardiovascular events (ACEs). However, there is a lack of data relative to large artery atherosclerosis (LAA) ischemic stroke. We investigated whether the monocyte to high-density lipoprotein (HDL) ratio (MHR) is related to the 3-month functional prognosis of LAA ischemic stroke.

**Materials and Methods:** A retrospective analysis was conducted on 316 LAA ischemic stroke patients. The 3-month functional outcome was divided into good and poor according to the modified Rankin Scale (mRS) score. Multivariate logistic regression analysis was performed to evaluate the correlation between MHR and prognosis of ischemic stroke.

**Results:** The MHR level of poor functional outcome group was higher than that of the good functional outcome group [0.44 (0.3, 0.55) vs. 0.38 (0.27, 0.5), *P* = 0.025]. Logistic stepwise multiple regression revealed that MHR [odds ratio (OR) 9.464, 95%CI 2.257–39.678, *P* = 0.002] was an independent risk factor for the 3-month poor outcome of LAA ischemic stroke. Compared to the lower MHR tertile, the upper MHR tertile had a 3.03-fold increase (95% CI 1.475–6.225, *P* = 0.003) in the odds of poor functional outcome after adjustment for potential confounders. Moreover, a multivariable-adjusted restricted cubic spline (RCS) showed a positive close to a linear pattern of this association.

**Conclusion:** Elevated MHR was independently associated with an increased risk of poor 3-month functional outcome of patients with LAA ischemic stroke.

## Introduction

Stroke has emerged as a major burden of the healthcare system and a frequent focus of chronic disease prevention and control in China (1). Ischemic stroke accounts for ~85% of all strokes, and it has emerged as one of the leading medical and health care problems worldwide (2). Atherosclerosis, especially intracranial atherosclerosis, is a highly prevalent cause of ischemic stroke (3). Large artery atherosclerosis (LAA) ischemic stroke is a common type of ischemic stroke, according to the classification of the Trial of ORG 10172 Acute Stroke Treatment (TOAST) (4). Studies reported that inflammatory markers, or the potential inflammatory processes they represent played an important role in the development of atherosclerosis, plaque rupture, and ultimately, arterial thrombotic diseases (5, 6). Some novel biomarkers, including adiponectin and neurofilament light chain, have been found to be able to predict the prognosis of ischemic stroke at an early stage (7, 8). Recently, the monocyte to high-density lipoprotein (HDL) ratio (MHR) has been considered as a new inflammatory marker, that includes the protective mechanism and injury mechanism, and is closely related to atherosclerosis and thrombosis (9, 10). Monocytes/macrophages are types of cells that play a key role in releasing pro-inflammatory cytokines and participate in all stages of the inflammation process (11). They promote inflammatory response and reduce plaque stability by releasing pro-inflammatory cytokines, and thus cause complications such as plaque rupture and hemorrhage, and thrombosis (12, 13). After the ischemic stroke event, monocytes/macrophages were also found to be involved in stroke-induced inflammation and injury (14). On the contrary, HDL can reversely transport cholesterol in the plaque through the ABCA1 pathway and protect the integrity of endothelial cells by inhibiting the oxidation of low-density lipoprotein (LDL) (15). In addition, it reduces the adhesion of monocytes by inhibiting the expression of endothelial cell adhesion molecules and inhibits the differentiation of monocytes to macrophages, which results in a limited inflammatory response (16, 17).

Current studies indicated that MHR was of great value in assessing the severity of coronary heart disease and predicting the incidence of adverse cardiovascular events (ACEs), such as stent thrombosis and mortality (18–20). Recently, a study found that high MHR level was related to the poor functional prognosis of patients with acute ischemic stroke, especially the stroke subtype of LAA (21). Other studies based on patients with ischemic stroke showed that elevated MHR level was associated with high mortality but a low risk of hemorrhagic transformation (22, 23). Indeed, there are few studies on the correlation between MHR and ischemic stroke, especially LAA ischemic stroke.

In this study, we aimed to evaluate the correlation between MHR and 3-month functional prognosis in LAA ischemic stroke patients.

## Materials and Methods

### Study Population

Medical records of patients, who were admitted to the Second Affiliated Hospital of Wenzhou Medical University, with the diagnosis of LAA ischemic stroke between January 2018 and August 2019 were retrospectively collected. The diagnosis of LAA ischemic stroke was referred to the TOAST system (4).

**Figure 1:**
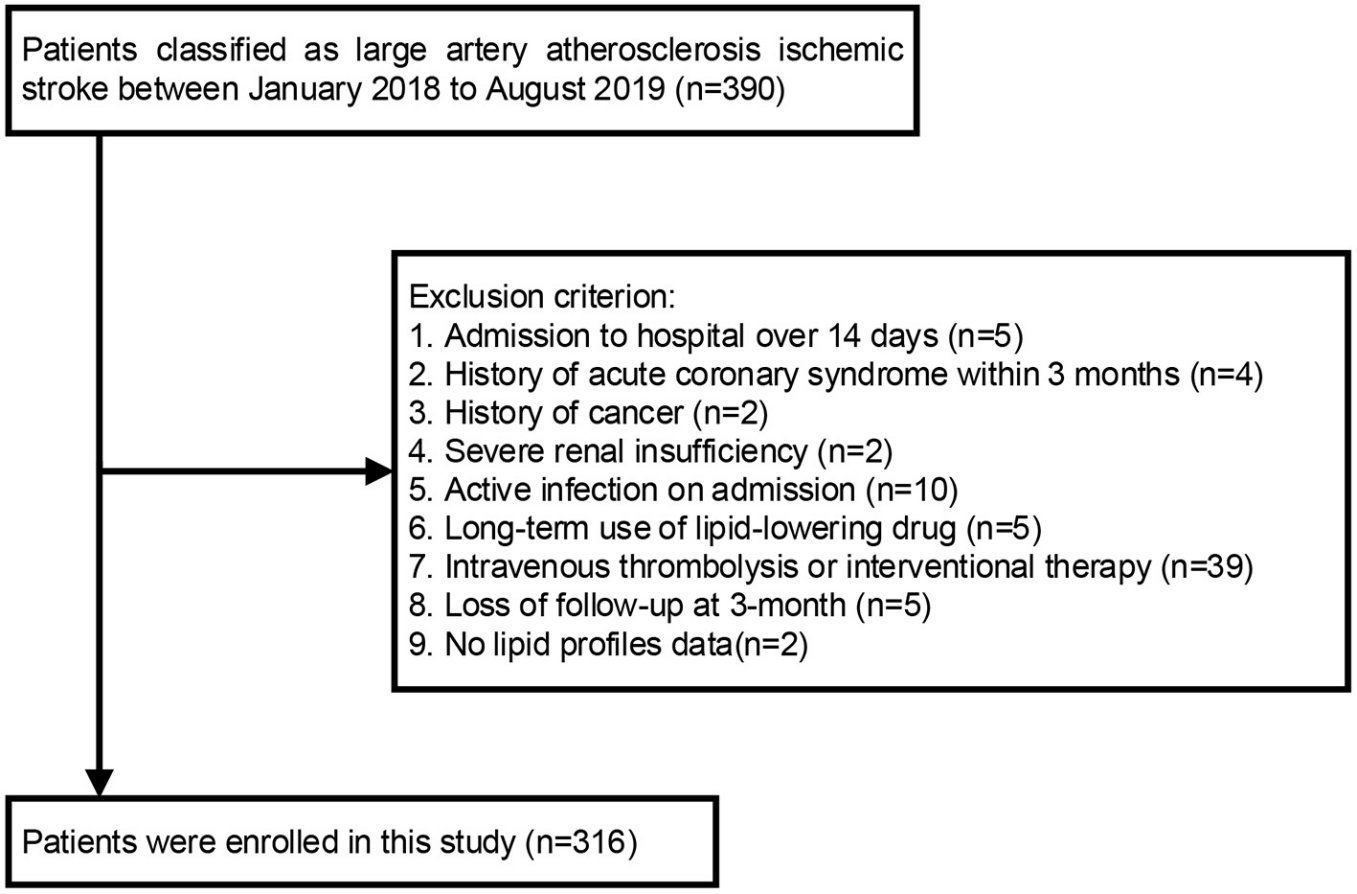
Flowchart of participant selection.

Exclusion criterion was as follows: (1) time from onset to admission over 14 days; (2) history of acute coronary syndrome (ACS) or stroke within the past 3 months, cancer, hematologic disorders, severe hepatic and renal insufficiency; (3) active infection on admission; (4) long-term use of the lipid-lowering drug (more than 1 month); (5) receiving intravenous thrombolysis or interventional therapy on admission; (6) incomplete medical records. Finally, a total of 316 patients were included in this study (Figure 1). This study protocol was approved by the Ethics Committee of the Second Affiliated Hospital and Yuying Children's Hospital of Wenzhou Medical University (approval no. 2021-k-100-01), and the whole data and procedures complied with the principle of ethical standards.

In this study, information about demographic and clinical characteristics, as well as hematologic and imaging data, were collected from medical records. Hypertension included systolic blood pressure (BP) ≥140 mmHg or diastolic BP ≥90 mmHg, or a previously diagnosed hypertension. Diabetes mellitus included fasting blood glucose (FBG) level ≥126 mg/dl (7 mmol/L), non-fasting glucose level ≥200 mg/dl (11.1 mmol/L), or a previously diagnosed diabetes. National Institutes of Health Stroke Scale (NIHSS) score was assessed by trained neurologists based on symptoms and signs at the time of admission. The 3-month functional prognosis was assessed according to the modified Rankin Scale (mRS). Good and poor functional prognoses were defined as mRS ≤ 2 and mRS >2, respectively. The time deviation of functional prognosis follow-up did not exceed 7 days.

### Laboratory Parameters

Venous blood samples were obtained within 24 h of admission. A hematological test was collected with an EDTA tube and biochemical analysis was collected with a dry tube. Cell counting and classification were performed using a Sysmex XE-5000 Automatic Hematology Analyzer (Sysmex Corp., Kobe, Japan). Biochemical parameters, including cholesterol level, were performed using a Beckman AU5800 Automatic Analyzer (Beckman Coulter Inc., CA, USA). MHR was calculated by dividing the monocyte count by the HDL cholesterol level.

### Statistical Analysis

Continuous variables were tested for normal distribution using Kolmogorov–Smirnov test. Normal distribution data were presented as mean ± *SD*, while non-normal distribution data were represented as medians (interquartile range, IQR). Independent-sample *t*-test and/or Mann-Whitney *U*-test were used for comparison between the two groups. Categorical variables were presented as numbers (percentages) and analyzed by chi-square or Fisher's exact test. Multivariate binary logistic regression analysis was performed to determine the independent risk factors for a 3-month poor prognosis of LAA ischemic stroke. Potential confounding covariates adjusted in multivariate logistic regression analyses included factors with a *P* < 0.1 in univariate analyses and other potential related factors. The continuous variable MHR was then divided into tertiles, and logistic analysis was conducted again to evaluate the correlation between MHR and 3-month functional prognosis. Finally, we used a multivariate logistic regression model with a restricted cubic spline (RCS) graph to explore the association between MHR and the risk of poor outcome of LAA ischemic stroke. Statistical analysis was performed using IBM SPSS Statistics version 26 (IBM Corp., Armonk, N.Y., USA) and R software, version 3.4.3 (R Foundation for Statistical Computing, Vienna, Austria). A two-tailed *P* < 0.05 was considered statistically significant.

## Results

### Baseline Characteristics

A total of 316 patients with LAA ischemic stroke were included in the analysis, including 209 males (66.1%) and 107 females (33.9%). The average age of the patients was 64.66 ± 12.24 years. In this study, the proportion of patients from onset to admission within 3 days was 81.3%. During hospitalization, 11 patients (3.5%) developed intracranial hemorrhage transformation (HT), 37 patients (11.7%) developed a nosocomial infection, and 6 patients (1.9%) developed deep venous thrombosis (DVT). During 3-month follow-up, 194 patients (61.4%) had good functional prognosis, and 122 patients (38.6%) had poor functional prognosis.

Baseline characteristics of patients between different outcome groups were summarized in Table 1. The MHR value of the poor outcome group was higher than that of the good outcome group, and the difference was statistically significant (*P* = 0.025). In addition, compared with patients with a 3-month good outcome, those patients with 3-month poor outcomes were older (*P* < 0.001), and comprised a higher frequency of diabetes mellitus (*P* < 0.001), higher baseline NIHSS score (*P* < 0.001) as well as a higher incidence of nosocomial infection (*P* = 0.04). Patients with poor outcome also had higher level of white blood cell (WBC) (*P* = 0.027), neutrophil (*P* = 0.015), and FBG (*P* = 0.004), but lower level of triglyceride (*P* = 0.025). The comparison results of other baseline indicators between the two groups were also shown in Table 1.

**Table 1:** Baseline characteristics of the study population in relation to 3-month outcome.

**Characteristics**	**Good outcome (*n* = 194)**	**Poor outcome (*n* = 122)**	***P***
Age, y, mean ± SD	62.29 ± 11.81	68.43 ± 11.99	<0.001^*^
Gender, male, *n* (%)	125 (64.4)	84 (68.9)	0.419
History of smoking, *n* (%)	70 (36.1)	35 (28.7)	0.174
History of drinking, *n* (%)	55 (28.4)	28 (23.0)	0.288
Hypertension, *n* (%)	171 (88.1)	113 (92.6)	0.199
Diabetes mellitus, *n* (%)	57 (29.4)	62 (50.8)	<0.001^*^
Systolic BP, mmHg, mean ± SD	158.05 ± 23.50	158.27 ± 24.87	0.937
Diastolic BP, mmHg, mean ± SD	86.05 ± 13.15	84.86 ± 13.61	0.440
NIHSS, median (IQR)	2 (1, 4)	7 (4, 12)	<0.001^*^
WBC, ×10^9^/L, median (IQR)	7.11 (5.59, 8.36)	7.55 (5.94, 9.48)	0.027^*^
Neutrophil, ×10^9^/L, median (IQR)	4.51 (3.47, 5.79)	5.02 (3.74, 7.08)	0.015^*^
Lymphocyte, ×10^9^/L, mean ± SD	1.82 ± 0.66	1.67 ± 0.72	0.052
Monocyte, ×10^9^/L, median (IQR)	0.40 (0.32, 0.51)	0.43 (0.32, 0.55)	0.103
Hemoglobin, g/L, mean ± SD	141.79 ± 15.68	140.20 ± 16.59	0.391
Platelet count, mean ± SD	228.63 ± 64.67	219.46 ± 65.97	0.224
FBG, mmol/L, median (IQR)	5.54 (4.82, 6.93)	6.47 (5.10, 8.86)	0.004^*^
Triglyceride, mmol/L, median (IQR)	1.48 (1.09, 2.08)	1.28 (0.96, 1.86)	0.025^*^
TC, mmol/L, mean ± SD	4.61 ± 1.08	4.7 ± 1.09	0.475
HDL, mmol/L, median (IQR)	1.04 (0.90, 1.27)	0.99 (0.82, 1.21)	0.079
LDL, mmol/L, mean ± SD	2.81 ± 0.96	2.96 ± 0.94	0.170
MHR, ×10^9^/mmol, median IQR)	0.38 (0.27, 0.50)	0.44 (0.30, 0.55)	0.025^*^
Infarction site, *n* (%)			0.244
Anterior circulation	120 (61.9)	86 (70.5)	
Posterior circulation	70 (36.1)	35 (28.7)	
Both	4 (2.1)	1 (0.8)	
HT, *n* (%)	4 (2.1)	7 (5.7)	0.156
Nosocomial infection, *n* (%)	17 (8.8)	20 (16.4)	0.040^*^
DVT, *n* (%)	1 (0.5)	5 (4.1)	0.065

*SD, standard deviation; IQR, interquartile range; BP, blood pressure; NIHSS, National Institutes of Health Stroke Scale; WBC, white blood cell; FBG, fasting blood glucose; TC, total cholesterol; HDL, high-density lipoprotein; LDL, low-density lipoprotein; MHR, monocyte to high-density lipoprotein ratio; HT, hemorrhagic transformation; DVT, deep vein thrombosis*.

* *P < 0.05*.

### Association Between MHR and Prognosis of LAA Ischemic Stroke

Age, gender, history of smoking, history of drinking, hypertension, diabetes mellitus, NIHSS, WBC, neutrophil, lymphocyte, FBG, triglyceride, HDL, MHR, nosocomial infection, and DVT were included in the multivariate analysis as independent variables. Logistic stepwise multiple regression revealed that age [odds ratio (OR) 1.045, 95%CI 1.02–1.071, *P* < 0.001], NIHSS (OR 1.357, 95%CI 1.249–1.475, *P* < 0.001), diabetes mellitus (OR 2.078, 95%CI 1.166–3.703, *P* = 0.013), MHR (OR 9.464, 95%CI 2.257–39.678, *P* = 0.002) were independent risk factors for the 3-month poor outcome of LAA ischemic stroke (Table 2).

**Table 2:** Multivariate logistic regression model^a^ of predictors to 3-month poor outcome.

**Variables**	**β**	**OR (95%CI)**	***P***
Age	0.044	1.045 (1.020–1.071)	<0.001^*^
NIHSS	0.306	1.357 (1.249–1.475)	<0.001^*^
Diabetes mellitus	0.732	2.078 (1.166–3.703)	0.013^*^
MHR	2.247	9.464 (2.257–39.678)	0.002^*^

*OR, odds ratio; CI, confidence interval; NIHSS, National Institutes of Health Stroke Scale; MHR, monocyte to high-density lipoprotein ratio*.

a *Age, gender, history of smoking, history of drinking, hypertension, diabetes mellitus, NIHSS, white blood cell, neutrophil, lymphocyte, fasting blood glucose, triglyceride, high-density lipoprotein, MHR, nosocomial infection, and deep vein thrombosis were included in the multivariate analysis as independent variables*.

* *P < 0.05*.

The MHR was then divided into tertiles (lower tertile: MHR ≥0.03, but <0.32; middle tertile: MHR ≥0.32, but <0.48; upper tertile: MHR ≥0.48, but ≤ 1.21). Univariate and multivariate logistic regression analyses were performed to analyze the association between MHR and 3-month functional prognosis. The crude OR of MHR in the upper tertile was 1.786 (95% CI 1.016–3.141, *P* = 0.044) compared with the lower tertile (Table 3). After adjustment for covariates, the upper tertile group remained a significant higher risk of poor outcome than lower tertile group (model 2: OR 1.865, 95%CI 1.016–3.422, *P* = 0.044; model 3: OR 3.03, 95%CI 1.475–6.225, *P* = 0.003; Table 3).

**Table 3:** Univariate and multivariate logistic analyses of the association between MHR and 3-month poor outcome.

**MHR tertiles**	**Model 1, unadjusted**		**Model 2** ^**a**^		**Model 3** ^**b**^	
	**OR (95%CI)**	***P***	**OR (95%CI)**	***P***	**OR (95%CI)**	***P***
Lower	1	–	1	–	1	–
Middle	1.298 (0.736–2.290)	0.368	1.031 (0.717–2.359)	0.387	1.935 (0.941–3.980)	0.073
Upper	1.786 (1.016–3.141)	0.044^*^	1.865 (1.016–3.422)	0.044^*^	3.030 (1.475–6.225)	0.003^*^

*OR, odds ratio; CI, confidence interval; MHR, monocyte to high-density lipoprotein ratio*.

a *Adjusted for age and gender*.

b *Adjusted for age, gender, history of smoking, history of drinking, hypertension, diabetes mellitus, National Institutes of Health Stroke Scale, white blood cell, neutrophil, lymphocyte, fasting blood glucose, triglyceride, high-density lipoprotein, nosocomial infection, and deep vein thrombosis*.

* *P < 0.05*.

**Figure 2:**
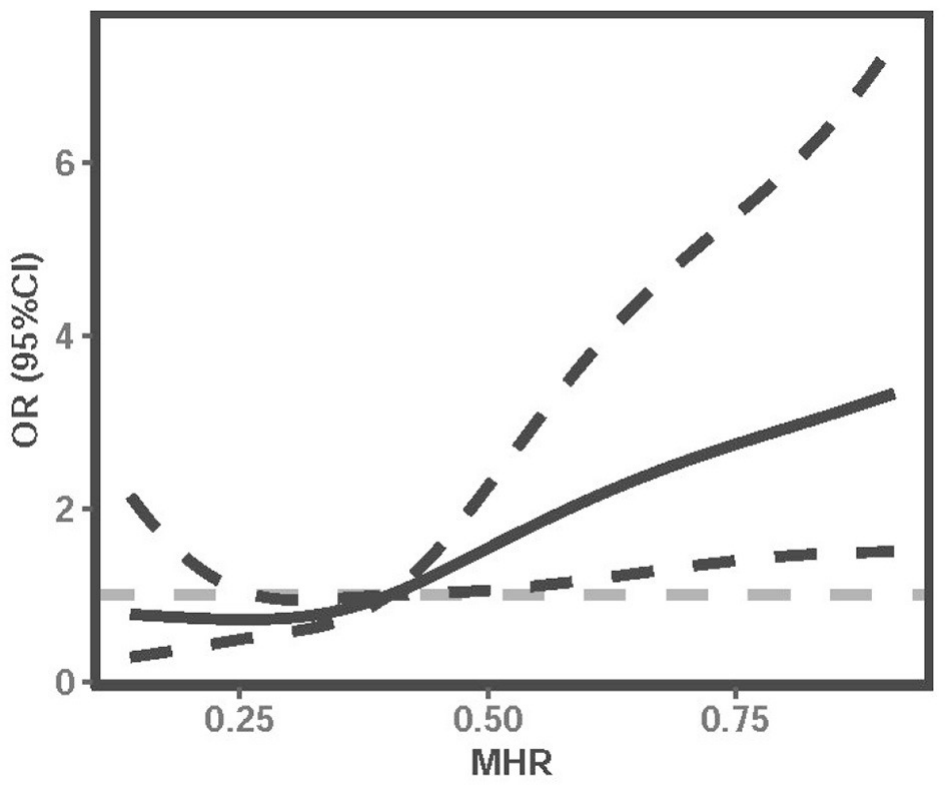
Multivariable-adjusted RCS analysis with four knots (at the 5th, 35th, 65h, 95th percentiles) was performed to explore the association between MHR and the risk of poor outcome of LAA ischemic stroke. It was adjusted for age, gender, history of smoking, history of drinking, hypertension, diabetes mellitus, NIHSS, WBC, neutrophil, lymphocyte, FBG, triglyceride, HDL, nosocomial infection, and DVT. The solid line indicates adjusted ORs, and the dotted line indicates 95%CIs. RCS, restricted cubic spline; OR, odds ratio; CI, confidence interval; MHR, monocyte to high-density lipoprotein ratio; NIHSS, National Institutes of Health Stroke Scale; WBC, white blood cell; FBG, fasting blood glucose; HDL, high-density lipoprotein; DVT, deep vein thrombosis.

In addition, we used multivariable-adjusted RCS to explore the dose-response relationship between MHR and the risk of 3-month poor outcome of LAA ischemic stroke (Figure 2). It showed that elevated MHR was associated with an increased risk of poor outcomes in LAA ischemic stroke patients.

## Discussion

This study was one of the few to explore the correlation between MHR and the prognosis of ischemic stroke. The main findings of this study were as follows: (1) LAA ischemic stroke patients with poor 3-month outcomes had a higher level of MHR than those with the good outcome; (2) after adjusting for potential confounders, MHR (OR 9.464, *P* = 0.002), age (OR 1.045, *P* < 0.001), NIHSS (OR 1.357, *P* < 0.001), and diabetes mellitus (OR 2.078, *P* = 0.013) were independent risk factors for the 3-month poor outcome of LAA ischemic stroke; (3) there was an approximate linear dose-response relationship between MHR level and the risk of poor functional outcome.

The most common etiological subtype of ischemic stroke in Asians is LAA, accounting for about 33%, while the prevalence of cardioembolism is significantly lower in Asians than in Caucasians (15 vs. 28%) (24). Wei and Quan analyzed protein-protein interaction (PPI) networks for all subtypes of ischemic stroke and indicated that focal inflammatory response, lipids storage dysregulation, and atherosclerotic plaque rupture were relevant to LAA ischemic stroke development (25). Further analyses from their study showed that the inflammatory pathway was the key etiology for LAA and lacunar subtypes, while FOS and JAK2/STAT3 signaling pathways might contribute to cardioembolism subtype (25). Interaction between inflammation and immunity involves various stages of atherosclerosis, which modulates lesion initiation, progression, and potentially devastating thrombotic complications (11). The process of ischemic stroke involves complex interactions among many cellular participants, including endothelial cells, the extracellular matrix, the blood-brain barrier (BBB), neurons, and the immune system (26). Inflammation involves various pathophysiological stages, which also amplifiers and propagate neuronal damage after ischemic injury (6, 26). Pro-inflammatory chemokines and cytokines such as nuclear factor kappa B subunit 1 (NFκB1), tumor necrosis factor -α (TNF-α), and interleukin-6 (IL-6) have been found to be important drivers of inflammatory responses in LAA type stroke (25).

The activation of monocytes is an important step in the initiation of atherosclerosis (27). Due to endothelial dysfunction and increased expression of cell adhesion molecules, monocytes aggregate, and enter the subendothelial space, where these cells differentiate into mononuclear phagocytes, ingest large amounts of normal and modified lipoproteins, which transforms them into cholesterol-laden foam cells (28). As a type of macrophages, foam cells are the main contributors to the inflammatory response in the processes of atherosclerosis. They secrete pro-inflammatory cytokines including TNF, IL-1, and IL-6, and chemokines such as CXC-chemokine ligand 1 (CXCL1), CC-chemokine ligand 2 (CCL2), and CCL5, as well as macrophage retention factors including semaphorin 3E and netrin 1, and they further release tissue factors and their lipid contents through their final death by necrosis or apoptosis, which causes the formation of a prothrombotic necrotic core (28). And this necrotic core is the crucial component of unstable plaques and contributes to their rupture and the following thrombosis that underlies acute coronary events and ischemic stroke (28, 29). The monocyte count in the circulating pool was also considered to be a predictor of new plaque formation (30). In addition, it was found that acute and prolonged inflammatory processes existed in the event of cerebral ischemia injury, including the rapid activation of resident immune cells (mainly microglia), production of pro-inflammatory factors, and infiltration of various inflammatory cells (including neutrophils, monocytes/macrophages, T cells, and other cells) into the ischemic tissue (31). Monocytes/macrophages were found to be involved in stroke-induced inflammation and injury (14). Kaito et al. found that the blood monocytes increased within 0–16 days after the occurrence of an acute ischemic stroke event, among which CD14(high)CD16(–) classical and CD14(high)CD16(+) intermediate monocytes increased significantly from 0 to 7 and 3 to 16 days after stroke, respectively, while CD14(+)CD16(high) non-classical monocytes decreased during 0–7 days after stroke (32). The typical monocyte corresponds to Ly6Chigh in rodents and has a significant pro-inflammatory effect, while the non-typical monocyte corresponds to Ly6Clow in rodents and has an anti-inflammatory effect (14, 32). The detection of monocyte subsets is complex and expensive, while blood monocyte count is easy to obtain. Therefore, clinical studies on the correlation between blood monocyte count and disease are more common. A high monocyte count level was found to be associated with a high risk of coronary heart disease and worse outcomes in ischemic stroke (33, 34). Moreover, it was revealed that elevated blood monocyte level was an independent risk factor for worse post-stroke functional outcomes after adjusting for associated risk factors (35). Contrary to the pro-inflammatory, promoting atherosclerosis, and promoting thrombotic properties of monocytes, HDL plays a reverse role, which has anti-inflammatory, anti-oxidant, and anti-thrombotic properties (36, 37). Taborda et al. indicated that HDL exerted an immunomodulatory effect by affecting the activity of innate immunosensors including dectin-1, Toll-like receptors (TLRs), and inflammasomes, and exerted an anti-inflammatory effect by reducing the production of IL-6 and IL-1β (38). Murphy et al. revealed that HDL and its major protein component apolipoprotein A-1 exhibit an anti-inflammatory effect on human monocytes by inhibiting the activation of CD11b (39). In the functional model, it was observed that cells adhered to an endothelial cell monolayer, and monocytes spread under shear flow and transmigrated (39). Additionally, evidence was found that HDL inhibited the uptake of LDL and modified LDL by mononuclear macrophages (40). Moreover, it was suggested that HDL could stimulate the reverse cholesterol transport process from foam cells in plaques to the liver, protect the endothelium by activating the endothelial nitric oxide synthase (eNOS) pathway, and inhibit oxidation of LDL (15). Recently, a retrospective study based on a large sample of 67,544 patients with type 2 diabetes found consistent inverse correlations between HDL level and the risk of ischemic and hemorrhagic stroke (41). The protective effect of HDL on cardiovascular disease also has been reliably quantified in the large prospective cohort (42). In addition, a combination of low HDL and high galectin-3 was found to be associated with poor prognosis after ischemic stroke, which includes the composite outcomes of death and vascular events, recurrent stroke, and vascular events (43). In recent years, MHR, as a new inflammatory marker, has been proved to have important value in coronary atherosclerotic heart disease. It was revealed that elevated MHR level, indicating an enhanced inflammation and oxidative stress, was significantly associated with the occurrence of slow coronary flow (18). Cetin et al. found that high MHR level was related to the occurrence of stent thrombosis in ST-elevation myocardial infarction (10). Moreover, a prospective large sample study of 3,798 patients undergoing coronary angiography showed that MHR, similar to monocyte were effective predictors of ACEs, including acute myocardial infarction, unstable angina, unexpected coronary revascularization, stroke, heart failure, and death (44). Similarly, studies have found that MHR can effectively predict the severity of the disease and the occurrence of ACEs in patients with ACS (20, 45). However, few studies previously focused on the role of MHR in ischemic stroke. In recent years, related studies have been gradually reported. A cross-sectional study estimated the association of MHR and prevalent ischemic stroke among a large cohort of the general Chinese population, which showed that individuals in the highest quartile of MHR level had a 1.6-fold higher risk of prevalent ischemic stroke compared with those in the lowest quartile, and MHR level was linearly associated with the incidence of ischemic stroke (46). Two studies based on patients who had undergone mechanical thrombectomy (MT) for large artery occlusion showed that elevated MHR was independently associated with a worse 3-month functional outcome, but not significantly associated with 3-month mortality (47, 48). Yuo et al. suggested that the presence of MHR did not impact long-term survival or stroke rate after carotid artery stenting (CAS) (49). Elevated MHR has also been found to be associated with a higher risk of HT in those patients undergoing MT (47). But another study showed that elevated MHR was associated with a lower risk of HT in ischemic stroke patients (22). In addition, a study on MHR and prognosis of acute ischemic stroke showed that non-surviving patients had higher MHR levels, and a high MHR value was an independent risk factor for 30-day mortality in patients with acute ischemic stroke (23). The study included all types of ischemic stroke, but unfortunately, the association between MHR and different subtypes of ischemic stroke was not analyzed. It is worth noting that the LAA subtype represented the highest proportion of patients enrolled in the study, accounting for about half of all patients (23). A recent study revealed that increased MHR level was associated with a higher rate of early neurological deterioration (END) in patients with isolated pontine infarction, which is considered a typical type of atherosclerosis-related infarction (50). Findings from Omar et al. showed that MHR was the independent predictor for the presence of carotid artery disease (occlusion, stenosis, or plaque) in ischemic stroke patients (51). These studies further reveal that there may be a close correlation between MHR and intracranial and extracranial atherosclerotic diseases. Moreover, another recent study similar to our study indicated that MHR was a risk factor for the 3-month functional prognosis of ischemic stroke (21). The subjects of their study included patients with all types of acute ischemic stroke within 24 h. A logistic model was used to analyze the relationship between MHR and prognosis in five different subtypes of ischemic stroke subgroups, and it was found that this conclusion only existed in LAA ischemic stroke subtype, and the OR value was 2.52 (21). This is similar to our results, the OR value of MHR in our model is 2.247. In addition, our study strengthened the control of confounding factors, such as the inclusion of infarct sites and common complications. In this study, although there were no differences in monocyte and HDL levels between the good outcome group and the poor outcome group, the MHR levels between the two groups were statistically different. We suggest that MHR may magnify the existed relationship between these two parameters and the prognosis of LAA ischemic stroke. Our study showed that MHR at admission was still an independent risk factor for the 3-month poor outcome of LAA ischemic stroke after adjusting for potential confounding factors. However, the exact mechanism between high MHR level and poor prognosis after ischemic stroke is still unclear, but as mentioned above, this index includes both protective and injury mechanisms, reflecting the degree of inflammation activation and vascular disease. In addition, our research showed that age, NIHSS, and diabetes mellitus were also independent risk factors for the 3-month prognosis of LAA patients. Similar to our study, age, NIHSS, and diabetes, especially NIHSS, have also been found in other studies to be related to the prognosis of patients with acute ischemic stroke (52, 53). New and useful markers that can early predict the prognosis of ischemic stroke are of great significance, which may become the new therapeutic target and even further improve the prognosis of patients. MHR may be expected to be a good and stable predictor of ischemic stroke. Indeed, this requires a lot of research to confirm in the future.

This study has several limitations. First, the present study was a single-center retrospective study, and the sample size is not large enough. Second, the inflammatory process has dynamics and continuity, but the MHR was not dynamically multiple times measured in our study. Therefore, the dynamic trend of the indicator and its value were not reflected in this study. Third, although other studies found that MHR was particularly closely related to LAA ischemic stroke, we did not conduct research in other ischemic stroke subtypes. For these reasons, more research is needed to further verify our findings and explore their underlying mechanisms.

## Conclusion

In conclusion, we suggest that elevated MHR value was dependently associated with an increased risk of the poor 3-month functional outcome in LAA ischemic stroke.

## Data Availability Statement

The raw data supporting the conclusions of this article will be made available by the authors, without undue reservation.

## Ethics Statement

The studies involving human participants were reviewed and approved by the Ethics Committee of the Second Affiliated Hospital and Yuying Children's Hospital of Wenzhou Medical University. Written informed consent for participation was not required for this study in accordance with the national legislation and the institutional requirements.

## Author Contributions

YL and WQ designed the study. DC, LS, and YL collected the data. ZC, WQ, and YL conducted the statistical analysis and manuscript writing. All authors read and approved the final manuscript.

## Conflict of Interest

The authors declare that the research was conducted in the absence of any commercial or financial relationships that could be construed as a potential conflict of interest.

## Publisher's Note

All claims expressed in this article are solely those of the authors and do not necessarily represent those of their affiliated organizations, or those of the publisher, the editors and the reviewers. Any product that may be evaluated in this article, or claim that may be made by its manufacturer, is not guaranteed or endorsed by the publisher.

## References

[1] Chao B Yan F Hua Y Liu J Yang Y Ji X. Stroke prevention and control system in China: CSPPC-Stroke Program. Int J Stroke. (2021) 16:265–72. 10.1177/1747493020913557 32223541

[2] Musuka TD Wilton SB Traboulsi M Hill MD. Diagnosis and management of acute ischemic stroke: speed is critical. CMAJ. (2015) 187:887–93. 10.1503/cmaj.140355 26243819 PMC4562827

[3] Banerjee C Chimowitz MI. Stroke caused by atherosclerosis of the major intracranial arteries. Circ Res. (2017) 120:502–13. 10.1161/CIRCRESAHA.116.308441 28154100 PMC5312775

[4] Adams HJ Bendixen BH Kappelle LJ Biller J Love BB Gordon DL. Classification of subtype of acute ischemic stroke. Definitions for use in a multicenter clinical trial. TOAST. Trial of Org 10172 in Acute Stroke Treatment. Stroke. (1993) 24:35–41. 10.1161/01.str.24.1.35 7678184

[5] van der Wal AC Becker AE van der Loos CM Das PK. Site of intimal rupture or erosion of thrombosed coronary atherosclerotic plaques is characterized by an inflammatory process irrespective of the dominant plaque morphology. Circulation. (1994) 89:36–44. 10.1161/01.cir.89.1.36 8281670

[6] Bonaventura A Liberale L Vecchie A Casula M Carbone F Dallegri F. Update on inflammatory biomarkers and treatments in ischemic stroke. Int J Mol Sci. (2016) 17:1967. 10.3390/ijms17121967 27898011 PMC5187767

[7] Tu W Qiu H Liu Y Liu Q Zeng X Zhao J. Elevated levels of adiponectin associated with major adverse cardiovascular and cerebrovascular events and mortality risk in ischemic stroke. Cardiovasc Diabetol. (2020) 19:125. 10.1186/s12933-020-01096-3 32771014 PMC7415178

[8] Nielsen HH Soares CB Høgedal SS Madsen JS Hansen RB Christensen AA. Acute neurofilament light chain plasma levels correlate with stroke severity and clinical outcome in ischemic stroke patients. Front Neurol. (2020) 11:448. 10.3389/fneur.2020.004489 32595585 PMC7300211

[9] Sercelik A Besnili AF. Increased monocyte to high-density lipoprotein cholesterol ratio is associated with TIMI risk score in patients with ST-segment elevation myocardial infarction. Rev Port Cardiol. (2018) 37:217–23. 10.1016/j.repc.2017.06.021 29615294

[10] Cetin EH Cetin MS Canpolat U Aydin S Topaloglu S Aras D. Monocyte/HDL-cholesterol ratio predicts the definite stent thrombosis after primary percutaneous coronary intervention for ST-segment elevation myocardial infarction. Biomark Med. (2015) 9:967–77. 10.2217/bmm.15.74 26439248

[11] Hansson GK Libby P Schonbeck U Yan ZQ. Innate and adaptive immunity in the pathogenesis of atherosclerosis. Circ Res. (2002) 91:281–91. 10.1161/01.res.0000029784.15893.10 12193460

[12] Hilgendorf I Swirski FK Robbins CS. Monocyte fate in atherosclerosis. Arterioscler Thromb Vasc Biol. (2015) 35:272–9. 10.1161/ATVBAHA.114.303565 25538208

[13] Weber C Shantsila E Hristov M Caligiuri G Guzik T Heine GH. Role and analysis of monocyte subsets in cardiovascular disease. Joint consensus document of the European Society of Cardiology (ESC) Working Groups “Atherosclerosis & Vascular Biology” and “Thrombosis”. Thromb Haemost. (2016) 116:626–37. 10.1160/TH16-02-0091 27412877

[14] Kim E Yang J Dbeltran C Cho S. Role of spleen-derived monocytes/macrophages in acute ischemic brain injury. J Cereb Blood Flow Metab. (2014) 34:1411–9. 10.1038/jcbfm.2014.101 24865998 PMC4126087

[15] Feig JE Feig JL Dangas GD. The role of HDL in plaque stabilization and regression: basic mechanisms and clinical implications. Coron Artery Dis. (2016) 27:592–603. 10.1097/MCA.0000000000000408 27414247 PMC5042826

[16] Navab M Reddy ST Van Lenten BJ Fogelman AM. HDL and cardiovascular disease: atherogenic and atheroprotective mechanisms. Nat Rev Cardiol. (2011) 8:222–32. 10.1038/nrcardio.2010.222 21304474

[17] Hoekstra M Van Eck M. Mouse models of disturbed HDL metabolism. Handb Exp Pharmacol. (2015) 224:301–36. 10.1007/978-3-319-09665-0_9 25522993

[18] Canpolat U Cetin EH Cetin S Aydin S Akboga MK Yayla C. Association of monocyte-to-HDL cholesterol ratio with slow coronary flow is linked to systemic inflammation. Clin Appl Thromb Hemost. (2016) 22:476–82. 10.1177/1076029615594002 26139836

[19] Ucar FM. A potential marker of bare metal stent restenosis: monocyte count – to- HDL cholesterol ratio. BMC Cardiovasc Disord. (2016) 16:186. 10.1186/s12872-016-0367-3 27716070 PMC5048646

[20] Karatas MB Canga Y Ozcan KS Ipek G Gungor B Onuk T. Monocyte to high-density lipoprotein ratio as a new prognostic marker in patients with STEMI undergoing primary percutaneous coronary intervention. Am J Emerg Med. (2016) 34:240–4. 10.1016/j.ajem.2015.10.049 26585199

[21] Liu H Liu K Pei L Gao Y Zhao L Sun S. Monocyte-to-high-density lipoprotein ratio predicts the outcome of acute ischemic stroke. J Atheroscler Thromb. (2020) 27:959–68. 10.5551/jat.51151 31941849 PMC7508725

[22] Wang Y Cheng Y Song Q Wei C Liu J Wu B. The association between monocyte to high-density lipoprotein ratio and hemorrhagic transformation in patients with acute ischemic stroke. Aging. (2020) 12:2498–506. 10.18632/aging.102757 32023223 PMC7041785

[23] Bolayir A Gokce SF Cigdem B Bolayir HA Yildiz OK Bolayir E. Monocyte/high-density lipoprotein ratio predicts the mortality in ischemic stroke patients. Neurol Neurochir Pol. (2018) 52:150–5. 10.1016/j.pjnns.2017.08.011 28864326

[24] Ornello R Degan D Tiseo C Di Carmine C Perciballi L Pistoia F. Distribution and temporal trends from 1993 to 2015 of ischemic stroke subtypes: a systematic review and meta-analysis. Stroke. (2018) 49:814–9. 10.1161/STROKEAHA.117.020031 29535272

[25] Wei LK Quan LS. Biomarkers for ischemic stroke subtypes: a protein-protein interaction analysis. Comput Biol Chem. (2019) 83:107116. 10.1016/j.compbiolchem.2019.107116 31561071

[26] Petrovic-Djergovic D Goonewardena SN Pinsky DJ. Inflammatory disequilibrium in stroke. Circ Res. (2016) 119:142–58. 10.1161/CIRCRESAHA.116.308022 27340273 PMC5138050

[27] Guermonprez P Helft J. Inflammasome activation: a monocyte lineage privilege. Nat Immunol. (2019) 20:383–5. 10.1038/s41590-019-0348-7 30858619

[28] Moore KJ Sheedy FJ Fisher EA. Macrophages in atherosclerosis: a dynamic balance. Nat Rev Immunol. (2013) 13:709–21. 10.1038/nri3520 23995626 PMC4357520

[29] Moreno PR Purushothaman KR Fuster V O'Connor WN. Intimomedial interface damage and adventitial inflammation is increased beneath disrupted atherosclerosis in the aorta: implications for plaque vulnerability. Circulation. (2002) 105:2504–11. 10.1161/01.cir.0000017265.52501.37 12034657

[30] Gratchev A Sobenin I Orekhov A Kzhyshkowska J. Monocytes as a diagnostic marker of cardiovascular diseases. Immunobiology. (2012) 217:476–82. 10.1016/j.imbio.2012.01.008 22325375

[31] Jin R Liu L Zhang S Nanda A Li G. Role of inflammation and its mediators in acute ischemic stroke. J Cardiovasc Transl. (2013) 6:834–51. 10.1007/s12265-013-9508-6 24006091 PMC3829610

[32] Kaito M Araya S Gondo Y Fujita M Minato N Nakanishi M. Relevance of distinct monocyte subsets to clinical course of ischemic stroke patients. PLoS ONE. (2013) 8:e69409. 10.1371/journal.pone.0069409 23936327 PMC3732285

[33] Olivares R Ducimetière P Claude JR. Monocyte count: a risk factor for coronary heart disease? Am J Epidemiol. (2006) 137:49–53. 10.1093/oxfordjournals.aje.a116601 8434572

[34] Bonifacic D Toplak A Benjak I Tokmadzic VS Lekic A Kucic N. Monocytes and monocyte chemoattractant protein 1 (MCP-1) as early predictors of disease outcome in patients with cerebral ischemic stroke. Wien Klin Wochenschr. (2016) 128:20–7. 10.1007/s00508-015-0878-4 26542133

[35] Liberale L Montecucco F Bonaventura A Casetta I Seraceni S Trentini A. Monocyte count at onset predicts poststroke outcomes during a 90-day follow-up. Eur J Clin Invest. (2017) 47:702–10. 10.1111/eci.12795 28783210

[36] Barter PJ Nicholls S Rye KA Anantharamaiah GM Navab M Fogelman AM. Antiinflammatory properties of HDL. Circ Res. (2004) 95:764–72. 10.1161/01.RES.0000146094.59640.13 15486323

[37] Karabacak M Kahraman F Sert M Celik E Adali MK Varol E. Increased plasma monocyte chemoattractant protein-1 levels in patients with isolated low high-density lipoprotein cholesterol. Scand J Clin Lab Invest. (2015) 75:327–32. 10.3109/00365513.2014.1003595 25797068

[38] Taborda NA Blanquiceth Y Urcuqui-Inchima S Latz E Hernandez JC. High-density lipoproteins decrease proinflammatory activity and modulate the innate immune response. J Interferon Cytokine Res. (2019) 39:760–70. 10.1089/jir.2019.0029 31335262

[39] Murphy AJ Woollard KJ Hoang A Mukhamedova N Stirzaker RA McCormick SP. High-density lipoprotein reduces the human monocyte inflammatory response. Arterioscler Thromb Vasc Biol. (2008) 28:2071–7. 10.1161/ATVBAHA.108.168690 18617650

[40] Carvalho MD Vendrame CM Ketelhuth DF Yamashiro-Kanashiro EH Goto H Gidlund M. High-density lipoprotein inhibits the uptake of modified low- density lipoprotein and the expression of CD36 and FcgammaRI. J Atheroscler Thromb. (2010) 17:844–57. 10.5551/jat.3905 20467189

[41] Shen Y Shi L Nauman E Katzmarzyk PT Price-Haywood EG Bazzano AN. Inverse association between HDL (high-density lipoprotein) cholesterol and stroke risk among patients with type 2 diabetes mellitus. Stroke. (2019) 50:291–7. 10.1161/STROKEAHA.118.023682 30626289 PMC6349480

[42] Di Angelantonio E Sarwar N Perry P Kaptoge S Ray KK Thompson A. Major lipids, apolipoproteins, and risk of vascular disease. JAMA. (2009) 302:1993–2000. 10.1001/jama.2009.1619 19903920 PMC3284229

[43] Zeng N Wang A Xu T Zhong C Zheng X Zhu Z. Co-effect of serum galectin-3 and high-density lipoprotein cholesterol on the prognosis of acute ischemic stroke. J Stroke Cerebrovasc Dis. (2019) 28:1879–85. 10.1016/j.jstrokecerebrovasdis.2019.04.007 31085131

[44] Zhang Y Li S Guo YL Wu NQ Zhu CG Gao Y. Is monocyte to HDL ratio superior to monocyte count in predicting the cardiovascular outcomes: evidence from a large cohort of Chinese patients undergoing coronary angiography. Ann Med. (2016) 48:305–12. 10.3109/07853890.2016.1168935 27087382

[45] Cetin MS Ozcan CE Kalender E Aydin S Topaloglu S Kisacik HL. Monocyte to HDL cholesterol ratio predicts coronary artery disease severity and future major cardiovascular adverse events in acute coronary syndrome. Heart Lung Circ. (2016) 25:1077–86. 10.1016/j.hlc.2016.02.023 27118231

[46] Wang HY Shi WR Yi X Zhou YP Wang ZQ Sun YX. Assessing the performance of monocyte to high-density lipoprotein ratio for predicting ischemic stroke: insights from a population-based Chinese cohort. Lipids Health Dis. (2019) 18:127. 10.1186/s12944-019-1076-6 31142338 PMC6542056

[47] Oh SW Yi HJ Lee DH Sung JH. Prognostic significance of various inflammation-based scores in patients with mechanical thrombectomy for acute ischemic stroke. World Neurosurg. (2020) 141:e710–7. 10.1016/j.wneu.2020.05.272 32522641

[48] Li X Wu F Jiang C Feng X Wang R Song Z. Novel peripheral blood cell ratios: effective 3-month post-mechanical thrombectomy prognostic biomarkers for acute ischemic stroke patients. J Clin Neurosci. (2021) 89:56–64. 10.1016/j.jocn.2021.04.013 34119295

[49] you TH Goodney PP Powell RJ Cronenwett J. “Medical high risk” designation is not associated with survival after carotid artery stenting. J Vasc Surg. (2008) 47:356–62. 10.1016/j.jvs.2007.10.046 18155875

[50] Bi X Liu X Cheng J. Monocyte to high-density lipoprotein ratio is associated with early neurological deterioration in acute isolated pontine infarction. Front Neurol. (2021) 12:678884. 10.3389/fneur.2021.678884 34262524 PMC8273253

[51] Omar T Karakayali M Yesin M Alaydin HC Karabag Y Gumusdag A. Monocyte to high-density lipoprotein cholesterol ratio is associated with the presence of carotid artery disease in acute ischemic stroke. Biomark Med. (2021) 15:489–95. 10.2217/bmm-2020-0705 33856263

[52] Rost NS Bottle A Lee JM Randall M Middleton S Shaw L. Stroke severity is a crucial predictor of outcome: an international prospective validation study. J Am Heart Assoc. (2016) 5:e002433. 10.1161/JAHA.115.002433 26796252 PMC4859362

[53] Luitse MJA Biessels GJ Rutten GEHM Kappelle LJ. Diabetes, hyperglycaemia, and acute ischaemic stroke. Lancet Neurol. (2012) 11:261–71. 10.1016/S1474-4422(12)70005-4 22341034

